# Reorganizing pediatric rehabilitation services to improve accessibility: do we sacrifice quality?

**DOI:** 10.1186/1472-6963-10-227

**Published:** 2010-08-05

**Authors:** Chantal Camden, Bonnie Swaine, Sylvie Tétreault, Marie-Michèle Brodeur

**Affiliations:** 1 Estrie Rehabilitation Center, Sherbrooke, Québec, Canada; 2 Center of Interdisciplinary Rehabilitation Research, Montréal, Québec, Canada; 3 École de réadaptation, Université de Montréal, Québec, Canada; 4 Institut de réadaptation Gingras-Lindsay de Montréal, Québec, Canada; 5 Centre de réadaptation Lucie-Bruneau, Montréal, Québec, Canada; 6 Département de réadaptation, Université Laval, Québec, Québec, Canada; 7 Institut de réadaptation en déficience physique de Québec, Québec, Québec, Canada; 8 Center for Interdisciplinary Research in Rehabilitation and Social Integration, Québec, Québec, Canada; 9 Université de Sherbrooke, Québec, Canada

## Abstract

**Background:**

The impact of a pediatric rehabilitation service delivery reorganization to improve access to services on parents' and service providers' perception of service quality was evaluated. Child-, family-, service- and service provider-related characteristics possibly associated with these perceptions were explored.

**Methods:**

Perceptions were measured using the Measure of Processes of Care tools and open ended questions before (2007), during (2008) and following (2009) service reorganization. Child and family characteristics, services received and service provider data were documented. Mean MPOC scores were compared over time (ANOVAs and Generalized Estimating Equations) and t-tests, correlations and ANOVAs determined whether the characteristics influenced scores.

**Results:**

Families' (n = 222) and service providers' (n = 129) perceptions of quality were high in 2007 (3.67 to 6.31/7) and remained high over the next 2 years (p ≥ 0.16). Two MPOC domain scores (Respectful care and Providing general information) were consistently scored the highest (mean ≥ 5.66/7) and the lowest (mean ≤ 4.75/7), respectively. Families with more education and those with children 12-21 years old tended to attribute lower MPOC scores. Participants were generally satisfied with the new service model and recommendations included improving information exchange.

**Conclusions:**

Results suggest that it is possible to reorganize pediatric rehabilitation services while maintaining quality.

## Background

Children with physical disabilities referred to rehabilitation services can face long delays before receiving these services [1-3]. This situation can affect families' stress, children's psychosocial well being and families' perception of service quality [2,4,5]. Despite these consequences, few concrete strategies have been presented in the literature to reduce waiting times for rehabilitation services. Examples include Clow & al. proposing 30-minute rather than 60-minute sessions to children with disabilities [6] and Sorel, Bouchard, Kalubi & Maltais who proposed evaluating children on the waiting lists, counseling their families and offering them information group sessions to foster family well-being while awaiting treatments [5]. Although interesting, these authors did not evaluate the impact of their temporary solutions on the overall quality of service provision (nor on patient outcomes).

Over the years, budget restraints and a growing need for services have led to an increase in delays for rehabilitation in the province of Québec, Canada [3]. Consequently, in September 2006, the pediatric rehabilitation program of the Eastern Townships Rehabilitation Center chose to develop and implement a new model of service delivery to address this issue. At the beginning of the reorganization process, many service providers feared that the strategies proposed to increase accessibility would negatively impact overall service quality [7]. Moreover, reorganization efforts may be destabilizing for service providers and may have a temporary negative impact on service quality [8-10]. Therefore, we conducted a study to determine whether the overall quality of services (as perceived by parents and service providers) was maintained during the reorganization process. We also explored whether child, family, service provider and service-related characteristics influenced these perceptions.

## Methods

### Setting and background

The rehabilitation program provides out-patient services to approximately 1000 families of children with various diagnoses aged 0-21 years living in the area. Children are treated within five subprograms listed in Table 1. Services were provided either at the center in the urban city, in one of the six rural rehabilitation settings or in the community.

**Table 1:** Characteristics of children and their families

Variables *	Total % (n = 222)	2007 % (n = 69)	2008 % (n = 80)	2009 % (n = 73)
Respondent				
Natural mother	82.0%	84.1%	82.5%	79.5%

Highest school degree completed by the respondent				
Collegial/University	52.3%	58.0%	52.5%	46.6%

Family situation				
2 parents	74.8%	73.9%	80.0%	69.9%

Language at home				
French	88.3%	87.0%	86.3%	91.8%

Area of residence				
Living in urban area	52.7%	62.3%	51.3%	45.2%

Family annual income				
35 000 or less CAN $	55.0%	49.3%	65.0%	49.3%

Age of child (years)				
Less than 5	24.3%	26.1%	25.0%	21.9%
5 to 11	57.7%	62.3%	50.0%	61.6%
12 to 21	18.0%	11.6%	25.0%	16.4%

Treatment sub programme				
Developmental delay	19.4%	22.1%	20.3%	16.4%
Dyspraxia	9.9%	10.3%	10.1%	9.6%
Motor	16.2%	17.6%	20.3%	11.0%
Speech and language	41.9%	41.2%	34.2%	52.1%
Teenager (multidisability)	11.7%	8.8%	15.2%	11.0%

Time since child began receiving services (years)				
	3.8 (SD:4.3)	3.8 (SD:4.3)	4.4 (SD:4.5)	3,2 (SD:4.0)

Hours of direct services received†	44.1 (SD:41.6)	40.3 (SD:45.5)	47.7 (SD:42.1)‡	43,6 (SD:37.2)

Total hours of service	94.7 (SD:86.0)	67.3 (SD:58.9)	115.1(SD:105.0)‡	98,0 (SD:78.4) ‡

Total hours of individual care	54.8 (SD:56.0)	67.1 (SD:59.0)	51.2 (SD:58.2) ‡	47,1 (SD:49.2) ‡

Total hours of group care	39.9 (SD:77.2)	0.2 (SD:1.3)	64.0 (SD:100.8) ‡	51,2 (SD:69.8)

SD: Standard Deviation.

* When only one category is presented, it means that this is a two-category variable;

† Hours of services received during the previous financial year (April 1^st ^to March 31);

‡ Significatively more hours of services than non participants (p value between 0.00 and 0.03);

Sum of percentages per category might be slightly under 100% due to missing data.

A planning committee, composed of administrators and a representative from each of the 6 disciplines within the program was formed to develop and to oversee the implementation of the new service delivery model. The service reorganization principally included new admission procedures and increased group and community interventions. The former included an initial contact with the family as soon as possible following referral to the centre, before treatment began. Specifically, a social worker telephoned or met with the families to discuss their priorities and the program's services (including community resources) and to determine the best services for their child. New group interventions (i.e. providing care to ≥ 2 children at a time) were developed within the centre and in the community and most involved more than one discipline. Groups were either diagnosis-specific (e.g. for children 4 years old with speech and language disorders) or goal-oriented. Most of the groups included children with different diagnosis (e.g. all children in the program to be enrolled in school). Community interventions targeting community partners in general rather than a particular group of children (e.g. general training for teachers about adapting to the needs of children with disabilities) were also developed.

### Participants and data sources

Two groups of subjects participated. The first group consisted of the parents of children aged 0 to 21 years old who received services from the program during the 2007, 2008 and 2009 fiscal years (ending on March 31). The second group included all the service providers working in the program at corresponding times over the study period. This group included the 6 representatives on the planning committee (from each of the program's disciplines) and the other service providers working in the program. About 50 service providers were working each year in the program, but some were missing during the data collection period (e.g. maternity leave) and could not complete the questionnaire.

In April of each year during the study period, eligible families (i.e. those receiving rehabilitation services for at least 6 months) were identified from the program's administrative database. This included approximately two thirds of the families listed in the program's database who were then grouped by area of residence (i.e. city center or one of the six adjacent regions) and a random sample, equal to one third of this size and stratified across the 7 areas, was created (about 300 families each year). The program's database contained information on the child's age and place of residence, the sub program in which he/she received services, when services began, and the numbers of hours and type of services (e.g. individual or group) received during the previous fiscal year. Hours of services included 1) Direct hours (i.e. provided in the presence of the child/family), 2) Total hours (including direct and indirect hours of services such as the time devoted to the preparation of the interventions etc.), 3) Total hours of individual care, and 4) Total hours of group care.

### Measurement tools

The Measure of Processes of Care (MPOC) [11] and the Measure of Processes of Care for Service Providers (MPOC-SP) [12] were used to evaluate the families' and service providers' perception of service quality, respectively. These tools provide a measure of the perception of the extent of family centeredness of rehabilitation services, a concept associated with perception of quality, satisfaction with services and family stress [13-16]. Both tools have good internal consistency and test retest reliability [11,12] and the MPOC has moderate 1-year stability [17]. French versions of the tools, back translated from the validated English versions and used in previous researches [2,18], were used in this study.

The MPOC consists of 56 questions grouped into five domains: 1) Enabling and partnership; 2) Providing general information; 3) Providing specific information about the child; 4) Coordinated and comprehensive care; 5) Respectful and supportive care. A sub score is calculated for each domain, representing the mean score for all items regrouped within this particular domain. Families are asked to respond on a 7-point scale how often each event happened to them during their episode of care (ranging from 0 'never' to 7 'always'). Although a validated shorter form exists (MPOC-20) [19], the MPOC-56 was chosen to allow a more detailed analysis and to identify areas of improvement to assist the program in the service reorganization process. For the purpose of this study, families were also asked the three things they liked most about the program and the three things they felt could be improved.

The MPOC-SP consists of 27 questions grouped into four domains (i.e. four subscores) varying only slightly from those of the MPOC: 1) Showing interpersonal sensitivity; 2) Providing general information; 3) Communicating specific information; 4) Treating people respectfully. Service providers are asked to respond on a 7-point scale 'in the past year, to what extent' they carried out a particular behaviour (from not at all to a great extent). They were also asked to rate their overall perception of service quality and the quality of the resources available in the program using a Likert scale (1-10, where 10 indicated completely satisfied). Finally, they were asked to indicate the three most important factors they felt positively influenced or hindered their ability to provide quality services, as well as their suggestions to improve the overall quality of services.

### Procedures

This study was part of a larger participatory action research that was approved by the ethics board of the Center of Interdisciplinary Rehabilitation Research of Montreal (CRIR-286-04-07). Each April, families were mailed a French or English version of the MPOC depending on their language of choice. Mailings also included a sociodemographic information sheet (relationship with child, respondent's highest academic degree, family situation (i.e. single parent), and family income), a pre-stamped return envelope and a consent form. Families were invited to complete and return the MPOC by mail or to call the center if they preferred to complete it over the phone. Telephone reminders were made at 1 and 3 weeks post mailing. Data from families who had participated in the study during a previous year were excluded such that we had 3 independent samples of parent data.

Almost all the service providers who were present at the annual program meeting held each Spring completed the French version of the MPOC-SP (only one or two each year either refused to participate or had to leave the meeting before completing the questionnaire). Service providers also provided professional information and written consent.

### Statistical and content analysis

Descriptive statistics (frequencies, means and standard deviations) were calculated for all variables. The characteristics of participating and non participating families were compared each year (t test and chi-square) with regards to the variables available in the program's administrative database. The characteristics of participating families were also compared across the three years using ANOVAs and chi-square (see variables listed in Table 1).

Mean scores were compared for the different domains across the three years using one-way ANOVAs and Generalized Estimating Equation (GEE) with the MPOC and the MPOC-SP data, respectively. The GEE procedure controls for the lack of independence among the samples [20] (i.e. the possibility that service providers could have responded once, twice or three times over the three-year data collection period). The results from the GEE procedure were similar to the results stemming from the ANOVAs for the MPOC-SP data. ANOVAs were also used to determine whether service providers' perception of the overall service quality and of the resources varied over time (i.e. scores on Likert scales).

Because the domains covered by the MPOC and MPOC-SP are not exactly the same, statistical comparison of the data from the two groups was not possible. For both sets of scores, however, we were able to identify the items for which 33% or more were rated «4» or less (occurring only 'sometimes') by respondents, thus ascertaining possible areas for improvement [21].

Bivariate analysis (t-test and correlations) and one-way ANOVAs examined the influence of child-, family and service provider-related variables on the mean scores for the different domains of the MPOC and MPOC-SP. Pearson correlations were performed with the number of hours of services received and the MPOC scores. T-test compared MPOC scores among those who had received group treatments compared to those who had not.

A thematic content analysis [22] was used to examine participants' answers to the open-ended questions. A coding grid inspired from the model of organized action system [23] and previously used in the larger participatory action research project, was used. Codes included structure (e.g. service organization and physical environment), actors (e.g. characteristics and practices of service providers), environment (e.g. community partners), processes (e.g. groups and information), impact (e.g. social participation), services (e.g. quantity of hours of services and accessibility), the reorganization of services (e.g. implementation of the new model and leadership) and the research process (i.e. perceptions about the participatory action research). Two researchers coded the verbatim and validated their coding by revising each other codes. Disagreements in coding were discussed until a consensus about the most appropriate code(s) was reached. Citations presented in this article were translated from French.

## Results

Over the three years, 222 of a possible 904 families participated in the study (2007, n = 69/223, 2008, n = 80/306, 2009 n = 73/375) for an overall participation rate of 24.6%. Analyses performed each year found no differences between responding and non responding families, but respondents tended to have received more hours of services than non respondents in 2008 and 2009. Generally, the characteristics of families were comparable across the years except for their annual revenue (χ^2 ^= 6.12, ddl = 2, p = 0.05); a greater percentage of them had a revenue < 35 000 CAN$ in 2008 (Table 1). Children received more total hours of services in 2008 (p = 0.02) and also tended to receive more in 2009 (p = 0.07) compared to 2007, but there were no significant differences in the number of direct hours of services received across the years. In 2008 and 2009, children received more group hours than in 2007 (p = 0.00), but the number of individual hours received did not vary significantly.

One hundred and twenty-nine questionnaires were completed by the service providers over the three years of this study (see Table 2) and the characteristics of the group were stable over time since the majority of them participated in the three data collections.

**Table 2:** Characteristics of service providers

Variables*	Total (n = 129)	2007 (n = 41)	2008 (n = 44)	2009 (n = 44)
Age of service provider				
≥ 36 years	73 (56.6%)	26 (63.4%)	24 (54.5%)	23 (52.3%)

Work experience (rehabilitation)				
≤ 10 years	66 (51.2%)	18 (43.9%)	23 (52.5%)	25 (56.8%)

Work experience (Centre)				
≤ 10 years	75 (58.1%)	22 (53.7%)	28 (63.6%)	25 (56.8%)

Work experience (paediatrics)				
≤ 10 years	69 (53.5%)	21 (51.2%)	25 (56.0%)	23 (52.3%)

Discipline				
Speech and language therapist	35 (27.1%)	11 (26.8%)	11 (25.0%)	13 (29.5%)
Occupational therapist	34 (26.4%)	12 (29.3%)	13 (29.5%)	9 (20.5%)
Special educator	26 (20.2%)	8 (19.5%)	9 (20.5%)	9 (20.5%)
Physical therapist	19 (14.7%)	6 (14.6%)	5 (11.4%)	8 (18.2%)
(Neuro)Psychologue/Social worker	15 (11.6%)	4 (9.8%)	6 (13.6%)	5 (11.4%)

Work status				
Part time	68 (52.7%)	22 (53.7%)	26 (59.1%)	20 (45.4%)

* When only one category is presented, it means that this is a two-category variable. Sum of percentages per category might be slightly under 100% due to missing data.

**Table 3:** Families' perception of the quality of rehabilitation services received as measured by the MPOC

MPOC domain	Mean scores (± SD)			ANOVA oneway (F, p value)
		
	2007 (n = 69)*	2008 (n = 80)	2009 (n = 73)	
Enabling and partnership	6.03 (1.13)	6.14 (0.79)	5.97 (1.05)	0.53, p = 0.59

Providing general information	4.68 (1.91)	4.73 (1.73)	4.75 (1.68)	0.02, p = 0.98

Providing specific information	5.78 (1.25)	5.89 (1.09)	5.80 (1.22)	0.13, p = 0.88

Coordinated and comprehensive care	6.05 (1.14)	5.99 (0.95)	5.86 (1.05)	0.59, p = 0.56

Respectful and supportive care	6.31 (0.80)	6.30 (0.69)	6.13 (1.00)	1.01, p = 0.37

SD: Standard Deviation;

* n varies for each domain within each year. A score can only be calculated for a domain if the respondent answers a minimum of 33% of the corresponding items.

Mean MPOC scores ranged from 4.68 to 6.31 and remained stable over the 3 years (p ≥ 0.37 for all domains). The domain Respectful and supportive care received the highest scores and the lowest scores were consistently attributed to Providing general information.

Mean MPOC-SP scores ranged from 3.67 to 5.89 and did not significantly differ over time (table 4) (p ≥ 0.16). Domains related to Respect and to General information were also rated the highest and the lowest, respectively.

**Table 4:** Service providers' perception of the quality of rehabilitation services provided as measured by MPOC-SP

MPOC-SP domain	Mean scores (± SD)			Wald Chi-Square (GEE) (p value)
		
	2007 (n = 41)*	2008 (n = 44)	2009 (n = 44)	
Showing interpersonal sensitivity	5.07 (0.58)	5.14 (0.61)	5.25 (0.81)	p = 0.47

Providing general information	3.67 (1.24)	3.89 (1.09)	4.08 (1.19)	p = 0.30

Communicating specific info. about the child	5.16 (0.93)	5.22 (1.07)	5.40 (1.04)	p = 0.46

Treating people respectfully	5.66 (0.60)	5.84 (0.51)	5.89 (0.64)	p = 0.16

SD: Standard Deviation. GEE: Generalized Estimating Equation.

* n varies for each domain within each year. A score can only be calculated for a domain if the respondent answers a minimum of 33% of the corresponding items.

Service providers' consistently rated the overall quality of services (F = 0.52, p = 0.60): 7.59, 7.38 and 7.47 out of 10 in 2007, 2008 and 2009, respectively. Their perception of the overall quality of resources was also stable over time (F = 1.93 and p = 0.15): 6.19, 5.74 and 6.41 out of 10 for the same years.

Bivariate analysis found that overall, only parents' education and child's age influenced the MPOC scores. Families with lower education scored higher on: Providing general information (p = 0.00), Providing specific information (p = 0.03) and Coordinated and Comprehensive Care (p = 0.04). Families with children 12-21 years old scored lower than those with children aged 5 to 11 years for Enabling and partnership (p = 0.03), Providing specific information (p = 0.05), Coordinated and comprehensive care (p = 0.05) and Respect and supportive care (p = 0.02). Hours of services received did not influence the MPOC domain scores (p ≥ 0.22) nor did the type of services (group or not) (p ≥ 0.15).

The service providers' characteristics listed in Table 2 did not generally influence MPOC-SP scores. Younger therapists (p = 0.00) and those with less rehabilitation (p = 0.01) or less pediatric experience (p = 0.03) scored higher on Communicating specific information about the child than those older and with more experience. Full time therapists attributed higher scores than those working part time for Treating people respectfully (p = 0.04) and those who were members of the planning committee scored higher than the others for Providing general information (p = 0.01). Regarding disciplines, special educators attributed higher scores than those of speech and language therapists for Showing interpersonal sensitivity (p = 0.04) and social workers and psychologists attributed higher scores than did occupational therapists (p = 0.02).

Tables 5 and 6 present the MPOC and MPOC-SP questionnaire items qualifying for areas for improvement. Over the three years, most items related to Providing general information and in general, the same items were highlighted year after year.

**Table 5:** Areas for improvement according to families' perceptions

Items identified as areas for improvement (scored ≤ 4 by > 33% of respondents)	% of scores ≤ 4 for the total sample (3 years)	Identified in 2007	Identified in 2008	Identified in 2009
**Providing General Information** (8/9 items identified at least once as areas for improvement in this domain)				

Q54. Provide advice on how to get information or to contact other parents (e.g., parent resource library at the centre)?	50.6%	√	√	√

Q49. Promote family-to-family gatherings for social, informational or shared experiences?	50.0%	√	√	√

Q50. Provide opportunities for special guests to speak to parents on topics of interest, if requested?	50.0%	√	√	√

Q46. Have information available to you in various forms, such as a booklet, kit, video, etc.?	44.4%	√	√	√

Q55. Provide opportunities for the entire family to obtain information?	39.4%	√	√	

Q56. Have general information available about different concerns (e.g., financial costs or assistance)?	38.4%	√	√	

Q48. Give you information about the types of services offered at the centre or in your community?	(32.1%)	√		

Q.51. Provide support to help cope with the impact of childhood disability (e.g., by advocating on your behalf or informing you of assistance programs)?	(31.3%)		√	

**Providing specific information about the child** (1/5 items identified at least once as areas for improvement in this domain)				

Q52. Notify you about the reasons for upcoming case conferences, meetings, etc. about your child?	38.5%	√	√	√

**Enabling and partnership** (0/16 items identified as areas for improvement in this domain)				

**Co-ordinated and comprehensive care** (0/17 items identified at least once as areas for improvement in this domain)				

**Respectful and supportive care** (0/9 items identified at least once as areas for improvement in this domain)				

**Table 6:** Areas for improvement according to service providers' perceptions

Items identified as areas for improvement (scored ≤ 4 by > 33% of respondents)	% of scores ≤ 4 for the total sample (3 years)	Identified in 2007	Identified in 2008	Identified in 2009
**Showing Interpersonal Sensitivity** (4/10 items identified at least once as areas for improvement in this domain)				

Q12. Help each family to secure a stable relationship with at least one service provider who works with the child and parents over a long period of time?	57.8%	√	√	√

Q11. Let parents choose when to receive information and the type of information they wanted?	50.4%	√	√	√

Q9. Anticipate parents' concerns by offering information even before they ask?	46.1%	√	√	√

Q8. Discuss/explore each family's feelings about having a child with special needs (e.g. their worries about their child's health or function)?	45.0%	√	√	√

**Providing general information** (5/5 items identified at least once as areas for improvement in this domain)				

Q26. Provide opportunities for the entire family, including siblings, to obtain information?	76.8%	√	√	√

Q23. Promote family-to-family "connections" for social, informational or shared experiences?	75.0%	√		√

Q25. Provide advice on how to get information or to contact other parents (e.g., through a community's resource library, support groups, or the internet)?	62.2%	√	√	√

Q27. Have general information available about different concerns (e.g., financial costs or assistance, genetic counseling, respite care, dating and sexuality)?	61.3%	√	√	√

Q24. Provide support to help families cope with the impact of their child's chronic condition (e.g., informing parents of assistance programmes, or counseling how to work with other service providers)?	44.8%	√		√

**Communicating Specific Information About The Child** (2/3 items identified at least once as areas for improvement in this domain)				

Q15. Provide parents with written information about their child's condition, progress, or treatment?	44.0%	√	√	√

Q16. Tell parents details about their child's services, such as the types, reasons for, and durations of treatment/management?	17.5%	√		

**Treating People Respectfully** (0/9 Items identified as areas for improvement in this domain)				

With regards to the responses to the open-ended questions, generally the same type of comments came back year after year. Two cross-cutting themes emerged from the codes: collaboration/information exchange and program characteristics. Data regarding these themes provided complementary information to that covered by the MPOC and MPOC-SP items.

Regarding parents' comments on collaboration and information, families appreciated the opportunity to discuss with service providers and to work toward the same goal. Families felt respected and considered the services providers as being competent: '*Service providers are doing what they can with the resources they have and they want our children to progress, they look for the best solutions*'. They also appreciated when rehabilitation teams partner with community members and follow up in the child's real life environments. Information exchange appears to be a key point in these partnerships. For example, families were happy not to have to repeat family information over and over again to different service providers. Families also appeared to want more information regarding the program's services (e.g. goals of the interventions and type of existing services) and the availability of community resources (e.g. respite care). Over the years, parents reported appreciating new features to improve information sharing that were introduced by the program, such as encounters for parents while children attend group treatments. Nonetheless, families would appreciate service providers being more proactive in offering information: '*We are often asked about our needs, how are we to know when you don't work in the field? They should make suggestions to us to get us to start thinking about what we might need*'.

Services providers also commented on collaboration and information, and discussed some organizational issues. Collaboration with families and community partners was perceived to be important: '*We should involve the parents even more, whenever possible, to counsel more and provide access to good information'*. Collaboration was nonetheless sometimes difficult to obtain for different reasons (e.g. families' characteristics, lack of resources or common goals with community partners, school schedule constraints, lack of time, heavy administrative procedures, etc). Community issues were highlighted more often in 2008-2009, probably reflecting an increase in community interventions. During the same timeframe, service providers reported that information sharing with families had improved over the years with the new admission procedures: '[*It enables us*] *to make rapid contact with the client and provide information'*. Still, service providers felt that families should be given more time to express their priorities, values and goals. Generally, service providers valued the provision of written information to families and wished to pursue the development of written information tools on services, diagnosis, general counseling, etc. They made suggestions about how to increase the program's efficiency: '[*We could] use the center's communication staff to develop information tools for families'*. Service providers also valued information exchange among themselves as it prevents duplication of work and fosters case discussion, sharing of intervention techniques and peer support. Moreover, they commented on their own need for information during the reorganization process and wanted quicker responses from their superiors on clinical or administrative issues. Staff turnover in leadership positions created insecurity among service providers and left things unanswered.

With regards to specific characteristics of the program services, many parents commented on the accessibility of services. Indeed, dissatisfaction with waiting times was mentioned year after year. One parent commented the following: '*Services given by intermittence [blocks of therapies interspersed with periods of no treatments] are a way to deal with waiting lists but are detrimental to our children'. *Nevertheless, parents appeared to be very satisfied with the quality of services once rehabilitation begins, but many would like more hours of services. A parent reported: '*Compared with the previous year, services are better. However, the duration of services is too short'*. Other parents commented on their desire to receive individual therapies and not only groups. A parent said: '*Services should respond to the real needs of the individual child - groups should not be used simply to reduce the waiting lists*'. Nonetheless, groups are much appreciated as they break isolation, foster expression and allow parents to observe their children: '*They provide my daughter the opportunity to be with children with similar problems. It is difficult to register her into groups with 'normal' children; teachers are not always patient... Your projects allow us to spend nice moments together!*'. In general, over the years, regardless of the type of services received, families reported that services were beneficial to the children as they helped them to become more autonomous, improve their self esteem and quality of life. Many stated that they particularly appreciated when services providers show them ways to work with their children and this helps them deal with daily issues.

Service providers also commented on the specific characteristics of the program services, and more specifically on the different type of services within the new service delivery model, its organizational aspects and the need to carefully plan services based on children's needs. The new admission procedures were seen as allowing them to see children sooner and younger, even if it's not always possible to respond to all of their needs: '[*We*] *get to know everyone on the waiting list and provide different interventions based on needs*'. Moreover, the increase in group and community interventions seemed to highlight more the organizational issues around service provision, such as the lack of time and the need for technical support (e.g. having someone to make appointments with families and prepare the materials). Service providers gave different suggestions to be more efficient: '*Simplifying forms, sharing assessment forms (...) to avoid repeating what was already done to be able to spend more time with clients*'. Services providers also reported feeling that a lack of resources contributed to limiting their ability to provide many hours of services of different types, often resulting in offering only group interventions to children. A service provider said: '[*When only groups are offered], it diminishes the quantity and quality of communication with the parents and thus limits our knowledge about the families and the children'*. Logistical issues with groups (e.g. schedule, materials) were also raised by service providers, but one person was encouraged that things were slowly but surely being put into place: '*Things should keep improving over time'*.

## Discussion

To our knowledge, this is the first study ever to examine the effect of broad organizational changes on pediatric rehabilitation service quality. MPOC and MPOC-SP scores were not significantly different over the three years, suggesting that the quality of services was likely preserved during the reorganization process. This is encouraging since organizational restructuring has been shown to temporally diminish service quality [8-10]. These results are even more interesting given that the pediatric rehabilitation program internal documents reported an increase of 32% in the number of children receiving rehabilitation services (from 972 in 2007 to 1279 in 2009) and a reduction of 41% in the number of children waiting for a first service (from 95 to 39 for the same period) [24]. Moreover, the number of hours of services received per child increased (total hours of services and group hours), indicating that the reorganization did not reduce the quantity of services provided. Accessibility issues seem to have been successfully addressed without sacrificing service quality.

The observed MPOC scores are similar or slightly superior to those reported in a 2006 review of studies using this tool to measure families' perception of service quality [25]. The lowest MPOC score reported was 3.61 for the Providing general information domain in the context of pediatric services in Britain [26] while the highest reported score was 6.66 for Respectful and supportive care in the context of an early intervention program in the United States [16]. Our MPOC scores are also similar to the only other study involving rehabilitation services in Québec in 1999 [18]. With regards to the MPOC-SP, the observed scores in the present study are generally similar or lower than those reported in the only three other studies using the MPOC-SP [12,16,18] presented in the review [25]. A more recent study in Québec used the MPOC-SP and reported similar scores to ours in year 1, although our scores by year 3 were higher than those reported in this other research [2].

Few studies have used the MPOC and MPOC-SP simultaneously. In this research, the MPOC-SP scores were consistently lower than the MPOC scores. This suggests that service providers are perhaps more critical than parents about the quality of care they provide to children and their families (e.g. they have an idea of optimal care whereas parents may not have a comparison base). Dyke et al found similar results [25], while others reported that service providers' scores compared to parents' scores were higher for some domains and lower for others [27,28]. Only one study using an adapted version of the MPOC with service providers reported service provider scores higher than those of families [18]. The variability in the MPOC and MPOC-SP scores reported is likely due to the broad diversity of settings in which the MPOC has been used.

In general, very few characteristics of the children, families, services and service providers measured in our study influenced the reported scores. These results are corroborated by some findings and conflicting with others in the literature. For instance, Raghavendra et al. (2007) reported that families' level of education influenced MPOC scores and that urban families tended to attribute lower scores than rural families for Providing general information. Moreover, our results showing that families with children 12-21 years old attributed similar scores to those with children 0 to 4 years old differ from those of several studies reporting that families with younger children (often preschoolers) tend to attribute higher scores [21,26,28-30]. We believe that our findings, showing only differences for families with children 5-11 years old, might be due to differences in the expectations regarding services as the child grows older [31] or a higher intensity of services provided to younger children [21]. However, we think it could also be due to differences in the services provided by the program before and after entering primary school, and after entering high school.

Service providers' age and experience as well as their discipline, their work status and participation in the planning committee for the service reorganization were found to influence some of the MPOC-SP scores. Interestingly, younger therapists tended to score higher on providing specific information. It is surprising considering that more experience is often associated with higher scores [2,25]. A possible explanation is the emphasis that has been put on information sharing in academic training in recent years; more recent graduates may have been more sensitized to these issues. The differences shown across disciplines could relate to differences in professional training, while being part of the planning committee exposed service providers to the emphasis that was put on information sharing during the meetings as part of the process of quality improvement.

Regarding service-related characteristics, some have previously argued that the MPOC can differentiate between different forms of service organization (e.g. among families having a care coordinator or not - [32], among children receiving a transition program or not - [26], or among families receiving services from different rehabilitation programs [33]). Until now, the influence of the intensity and the type of service (individual or group) has never been investigated. In our study, these variables were not found to be associated with MPOC scores, despite that many families reported wanting more hours of services and more individual services as well as those provided in a group. These are interesting results as intensity of services is seldom associated with better patients' outcomes [34].

The majority of items that qualified as areas for improvement in the present study are similar to those found by others [25]. Our study is however the first to present longitudinal follow up data for areas for improvement. Interestingly, some areas for improvement disappeared in years two and three, perhaps reflecting the increased awareness by the program of the importance of providing general information to families. Indeed, written materials and new services were developed during the reorganization to increase information sharing. Information kits [35] and having a key worker [36] or care coordinator [26] have been reported as strategies to improve service quality.

This study supports the use of MPOC and MPOC-SP tools to help measure the impact of organizational changes in rehabilitation service delivery. They have been used to evaluate specific modifications in a program or new resources and services [35,36], but this is the first time that both tools were used to follow up and document perceptions of quality of care during the implementation of a new pediatric rehabilitation service delivery model. Moreover, the additional open opened questions provide useful complementary information regarding themes not included in these tools. These questions raised issues that the program could target to improve service quality (e.g. recommendations regarding organizational issues to increase program's efficiency). One must remember that quality is a multidimensional concept that might be better targeted by the simultaneous use of different tools using mixed methods. The MPOC and MPOC-SP tackle specific aspects of service quality including how services are provided. New types of services such as groups and community interventions might change the dynamic between families and service providers, thus modifying the relationship components upon which the MPOC and the MPOC-SP are built. Future directions to consider in the context of evaluating the perception of the quality of pediatric rehabilitation services should incorporate more service-related variables, such as detailed intensity and type of services measures. Future studies should thus take into account characteristics of service organization and organizational culture. Perception of quality should also be evaluated alongside with a measure of progress of the child to better document the relation between perception of quality and children's outcomes'.

Study limitations include potential recall bias or social desirability, possibly explaining why families tended to score higher than service providers. Moreover, despite various reminders, our response rates for families are among the lowest reported in the literature; two studies report rates below 30% [35,37], while others have reported rates over 65% [26,28]. The type and the quantity of rehabilitation services received by the family in the previous year could influence families' willingness to respond. For instance, in our study, non-responding families tended to have received fewer services than respondents thus reducing their desire to participate in the study. Finally, multiple testing may have led to the numerous significant findings.

## Conclusions

The results suggest that it is possible to reorganize services to improve accessibility to pediatric rehabilitation services while maintaining service quality. Results also suggest that new service delivery models of pediatric rehabilitation should include features to increase information sharing with families and among interdisciplinary rehabilitation team members. This is especially true during service reorganization when the process can be destabilizing for families and service providers. Understanding and evaluating the multiple dimensions of quality in pediatric rehabilitation services is important to generate useful knowledge to help clinical settings in their continuous quality improvement efforts.

## List of abbreviations

MPOC: Measure of Processes of Care; MPOC-SP: Measure of Processes of Care for Service Providers.

## Competing interests

The authors declare that they have no competing interests.

## Authors' contributions

All authors made substantial contributions to the study conception and design and manuscript, and approved the final version of this manuscript. CC designed the study under the supervision of BS and ST, coordinated the data collection, performed and interpreted the analyses and wrote the first draft of the manuscript. BS and ST supervised the data collection and critically revised the manuscript. MMB conducted part of the data collection, participated in the analysis and interpretation of results and commented on the manuscript.

## Authors' information

This manuscript is a part of the doctoral thesis of the first author. The thesis is to be published under electronic form in the forthcoming year.

## Pre-publication history

The pre-publication history for this paper can be accessed here:

http://www.biomedcentral.com/1472-6963/10/227/prepub
